# Determinants of the Stunting of Children Under Two Years Old in Indonesia: A Multilevel Analysis of the 2013 Indonesia Basic Health Survey

**DOI:** 10.3390/nu11051106

**Published:** 2019-05-18

**Authors:** Christiana R. Titaley, Iwan Ariawan, Dwi Hapsari, Anifatun Muasyaroh, Michael J. Dibley

**Affiliations:** 1 Faculty of Medicine, Pattimura University, Kampus Poka, Maluku Province, Ambon 97233, Indonesia; 2 Center for Health Research, Faculty of Public Health Universitas Indonesia, West Java Province, Depok 16424, Indonesia; ariawan@dnet.net.id (I.A.); anifatun.muasyaroh@gmail.com (A.M.); 3 National Institute of Health Research and Development, Ministry of Health Republic of Indonesia, DKI Jakarta 10560, Indonesia; dhapsari1971@gmail.com; 4 School of Public Health, The University of Sydney, Sydney, NSW 2006, Australia; michael.dibley@sydney.edu.au

**Keywords:** stunting, child under two years old, Indonesia, Indonesia Basic Health Survey

## Abstract

Indonesia is ranked fifth among countries with the highest burden of stunting in children under five. This study aims to examine the determinants of stunting in children aged 0–2 years in Indonesia using data derived from the 2013 Indonesia Basic Health Survey. Twenty potential predictors of stunting, categorized into household and housing characteristics; maternal and paternal characteristics; antenatal care services and child characteristics were analyzed. Multilevel analyses were performed to examine the role of cluster/district/provincial differences, as well as individual/household level characteristics and stunting status. Of 24,657 children analyzed, 33.7% (95%CI: 32.8%–34.7%) were stunted. The odds of stunting increased significantly among children living in households with three or more children under five-years-old (aOR = 1.33, 95%CI: 1.03–1.72), households with five to seven household members (aOR =1.11; 95%CI: 1.03–1.20), children whose mothers during pregnancy attended less than four antenatal care services (aOR = 1.22, 95%CI: 1.08–1.39), boys (aOR = 1.33, 95%CI: 1.22–1.45), children aged 12–23 months (aOR = 1.89; 95%CI: 1.54–2.32), and children who weighed <2500 g at birth (aOR = 2.55; 95%CI: 2.05–3.15). The odds also increased significantly with the reduction of household wealth index. Integrated interventions to address environment, an individual level associated with stunting in Indonesia, from the environment- to individual-level factors are important.

## 1. Introduction

Stunting or poor linear growth (height-for-age-Z score ≤−2) is considered to be a major public health problem among children globally [1,2]. Approximately 151 million (22%) children under five-years-old in 2017 were affected by stunting [3]. More than half of children with stunting are from Asia [3].

Stunted children are affected by poor nutrition in-utero and early childhood, as well as frequent infections before or after birth and therefore have a greater risk for illness and death [3,4,5]. Research shows that stunted children may never reach their full potential height and have poor cognitive development leading to suboptimal educational performance and reduced intellectual capacity, motor and socioeconomic development [3,4,5]. Additionally, stunted women are at greater risk for developing obstetric complications due to a smaller pelvis, delivering low birth weight infants resulting in an increased risk of chronic non-communicable diseases in adulthood, as well as a cycle of malnutrition, as low birth weight infants are more likely to be smaller as adults [5].

The critical consequences of stunting have led to the setting of global nutrition targets to reduce the number of stunted children under five by 40% by 2025 [6]. This global target has since been further supported by the Sustainable Development Goal 2, target 2: “*By 2030, end all forms of malnutrition, including achieving, by 2025, the internationally agreed targets on stunting and wasting in children under 5 years of age, and address the nutritional needs of adolescent girls, pregnant and lactating women and older persons.*” [7].

According to the World Health Organization (WHO)’s cut-off values of public health significance for stunting, Indonesia is considered to have a high prevalence of stunting (30–39%) [5]. The country is even ranked fifth among countries with the highest burden of stunted children [8]. The reduction of stunting prevalence has been slowly progressing in the last ten years, from 42% to 36% [8]. The 2013 Indonesia Basic Health Survey reported that around 37.2% of children under five years in Indonesia are stunted, ranging from around 27% in Kepulauan Riau Province to >50% in Nusa Tenggara Timur Province. This study, therefore, aimed to examine determinants of stunting in children aged 0–2 years in Indonesia using the 2013 Indonesia Basic Health Survey.

## 2. Materials and Methods

### Data Source and Survey Design

This analysis used data derived from the 2013 Indonesia Basic Health Survey conducted by the National Institute of Health Research and Development, Ministry of Health, Republic of Indonesia. It is a five-yearly cross-sectional survey conducted by the Ministry of Health since 2007 that collects basic information and health-related indicators that depict the health situation of the community at the district/city, provincial and national levels. The main objectives of the survey were to assess the achievement of community health status at the district/city, provincial and national levels and to detect any specific changes of health problems at those levels [9].

The 2013 Indonesia Basic Health Survey survey included households from all 33 provinces and 497 districts/cities of Indonesia. There were 11,986 census blocks visited of 12,000 census blocks targeted (99.9%); 294,959 households visited, and 1,027,763 household members interviewed with a response rate of 93.0%. There were two types of structured questionnaires used, namely the individual and household questionnaire. Detailed explanation about the survey methodology has been described in detail elsewhere [10]. In this analysis, we used information collected from 24,657 women with children under two years of age.

## 3. Variables

**Figure 1:**
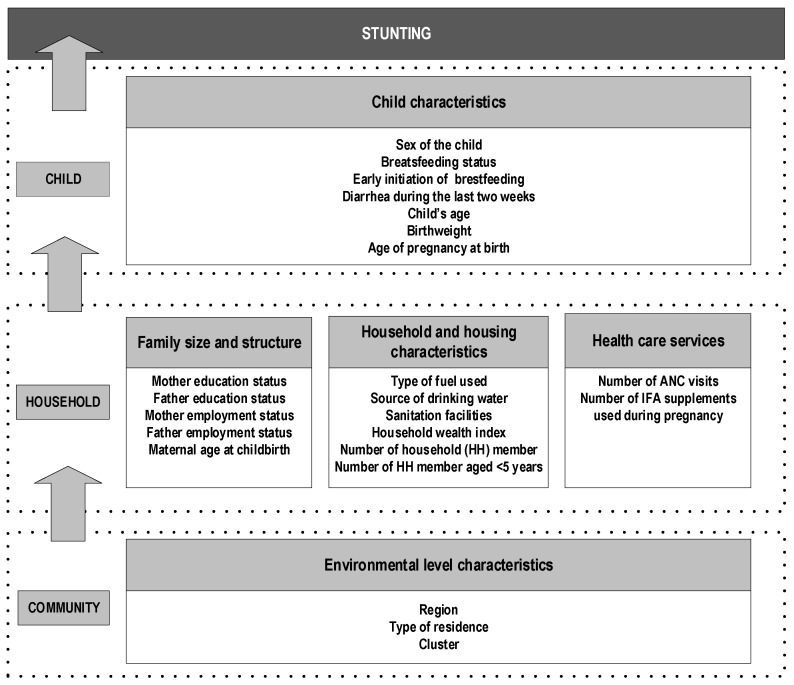
An analytical framework for factors associated with stunting among children under two years of age in Indonesia.

The theoretical framework developed by the WHO [11] was adapted to assess the relationship between different potential predictors available in the survey dataset and stunting among children under two-years-old (Figure 1).

### 3.1. Outcome Variable

The primary outcome of this analysis is low height-for-age (stunting) of the child measured at the time of the survey. In this survey, the body length of children was measured using a customized multifunction stadiometer designed specifically for this survey. This tool could be used to measure both adult height and baby length. Its maximum measuring capacity for children was 135 cm and an accuracy of 0.1 cm. For children aged <2 years, recumbent length was used [12]. Height-for-age was the difference between the child’s measured height and the mean height of healthy children in the same age and sex group, expressed as the number of standard deviations or Z-score. We classified a child as being stunted if their height-for-age Z-score (HAZ) was less than −2.2 [13], and assigned a score of 1 to stunted children, and 0 to those not stunted.

### 3.2. Potential Predictors

In total, we analyzed 20 potential predictors of stunting, categorized into four main groups, i.e., household and housing characteristics; maternal and paternal characteristics; antenatal care services and child characteristics (Figure 1). There were six variables under the household and housing characteristics, i.e., the number of all household members, the number of children under five years in the household, the type of fuel used for cooking, the source of drinking water, sanitary facilities at home and household wealth index, which was constructed as a proxy for household economic status. We created this variable from an inventory of household facilities and assets using the Principal Component of Analysis [14]. We then ranked all households according to the household wealth index and formed five quintiles; i.e., poorest, poor, middle, rich and richest households.

In the maternal and paternal characteristics group, five variables were included; i.e., education status of mother and father; the employment status of mother and father, as well as maternal age at childbirth. For the antenatal care services group, we used the number of antenatal care visits and the number of iron/folic acid supplements used during pregnancy. In the child’s characteristics group, there were seven potential predictors included, which were child’s sex, child’s weight at birth, age of pregnancy at birth, ever breastfed, time to initiate breastfeeding after birth, history of diarrhea during the past two weeks and child’s age at the time of interview.

### 3.3. Data Analysis

At the initial stage, descriptive statistics were employed to examine all variables used, followed by bivariate analysis to examine their distribution by stunting status. Logistic regression analyses were then conducted for each potential predictor to determine the unadjusted odds ratio (OR) as the estimated measures of association between outcome variables and their potential predictors. In the next stage, we performed the multilevel analysis and two sequential models, including random intercepts.

Firstly, we constructed a Null model (empty model) aimed at assessing the role of the cluster, district, and province without adjusting for region and all potential predictors at the individual/household level. The median odds ratio (MOR) was calculated for each level to measure its association with stunting status.

Secondly, Model 1 was developed to examine the role of cluster/district/provincial differences as well as individual/household level characteristics and stunting status, after adjusting for one another. The measure of association used in this model was the aOR (adjusted odds ratio). A backward elimination method was employed to remove all individual/household level characteristics not significantly related to the study outcome, using the significance level of 0.05 while the cluster, district and province variables remained in the models. We obtained all aORs and 95% confidence intervals (95%CIs) for all predictors retained in the final model. All estimates presented in this analysis considered the complex sample design. The statistical analysis on the data was carried out with the use of Stata/MP software (version 14.2; StataCorp, College Station, TX, USA) using xtmelogit routine.

### 3.4. Ethics Clearance

Ethics clearance to conduct the 2013 Basic Health Research was obtained from the Ethics Committee of National Institute of Health Research and Development, Ministry of Health, Republic of Indonesia.

## 4. Results

Our results showed that of 24,657 children under two-years-old included in this study, 33.7% (95%CI: 32.8–34.7%) were stunted. As shown in Table 1, the rate of stunting was significantly different (*p* = 0.01) across regions. Generally, the rate was higher in the eastern than western parts of Indonesia. The region of Nusa Tenggara Barta and Nusa Tenggara Timur (NTB/NTT) had the highest stunting rate (42.3%), while the lowest was in the Java-Bali Region (31.7%).

The distribution of all respondents by household and housing, maternal and paternal, antenatal care services and child characteristics are presented in Table 1. Without adjusting for any other covariates, there was a significant association between stunting and region, number of household members; number of household members under five years; type of fuel used for cooking; unimproved sanitary facility; household wealth index; mother’s and father’s education status; mother’s age at childbirth; number of ANC visits, child’s sex; child’s age at the time of interview; child’s weight at birth and mother’s age of pregnancy at birth.

The results of multilevel modeling are presented in Table 2. The Null model shows that the MOR of district and province was 1.32 and 1.22, respectively. A stronger effect of the cluster was reflected by the high MOR (3.27). When individual-level factors (household, maternal/paternal, antenatal care and child variables) were added into the null model (Model 1), all MORs changed, but only slightly. The province’s MOR reduced by 14%, the district’s MOR increased slightly by 1.5% and the cluster’s MOR increased by only 1.2%. These findings mirror the consistent role of the province, district and cluster even after the inclusion of individual-level variables in the model. Furthermore, this analysis found that the residual heterogeneity between clusters (MOR = 3.27) was of greater relevance than was the other individual-level variables on stunting.

In our Model 1 analysis we found significantly higher odds of stunting for children under two from outside the Java and Bali region. At the household level, the odds of stunting increased significantly among children living in households with three or more children under five-years-old (aOR = 1.33, 95%CI: 1.03–1.72, *p* = 0.029). Also, the odds increased in children from households where five to seven members lived (aOR = 1.11; 95%CI: 1.03–1.20, *p* = 0.005). The odds of stunting significantly increased along with the reduction of the quintile of the household wealth index (Table 2). Children of mothers attending less than four ANC were more likely to be stunted than those whose mother attended four or more ANC (aOR = 1.22, 95%CI: 1.08–1.39, *p* = 0.002). Boys had 33% higher odds of being stunted than girls (aOR = 1.33, 95%CI: 1.22–1.45, *p* < 0.001), and children aged 12–23 months had 89% higher odds of being stunted than those aged <12 months (aOR = 1.89; 95%CI: 1.54–2.32, *p* < 0.001). The odds of stunting in children who weighed <2500 g at birth was 2.55 times the odds of children weighing ≥2500 g (aOR = 2.55; 95%CI: 2.05–3.15, *p* < 0.001).

## 5. Discussion

### 5.1. Main Findings

We found that the odds of stunting increased significantly among children living in households with three or more children under five-years-old, households with five to seven household members, children whose mother during pregnancy attended less than four antenatal care services, boys, children aged 12–23 months and children who weighed <2500 g at birth. Also, the odds of stunting increased significantly along with the reduction of household wealth index. Our findings using nationally representative data should be of interest to policy makers and relevant stakeholders at the national level to help design effective evidence-based interventions to reduce the prevalence of stunting amongst children under two-years-old in Indonesia.

### 5.2. The Role of Community and Household Level Variables

Our study found that region and cluster where mothers lived were among the significant predictors of stunting in Indonesia as reported in other studies [15,16]. In our analysis, children from outside Java-Bali areas, as predicted, had an increased likelihood of stunting compared to those living in Java-Bali areas. This condition might reflect a lower socio-economic condition of communities in the outer Java Bali areas, particularly those in the eastern part of Indonesia. Studies have shown that there are more limited resources and facilities, including health care personnel and services in outer Java Bali compared to the Java-Bali region [17,18]. The high proportion of households in the outer Java-Bali region with limited access to improved latrines might be among the factors contributing to a higher prevalence of stunting in the region [19].

At the household level, we found an association between household wealth index and stunting. Higher wealth index reflects an increased ability of a household to purchase and access good quality food and adequate health care services, as well as improved sanitation facilities and safe drinking water. Appropriate hygienic practices have been reported to potentially improve child growth through the prevention of various morbidities [20,21]. Also, the relationship between low household wealth index and stunting might work through the food insecurity status of the household [22,23] and fulfillment of minimum dietary diversity in children [22,24]. Households with high household wealth index are more likely to be food secure and able to meet a child’s dietary needs.

Other significant factors at the household level found in this analysis were family size and number of children under five years living in the household. Improper allocation of food and other resources in households with many children can lead to their poor health and sub-optimum nutritional status. Furthermore, a large household might suggest resource depletion, reduced food availability, accessibility and competition for scarce resources [25,26,27,28]. The presence of more that one child under five-years-old could also result in sub-optimal breastfeeding and complementary feeding practices.

These findings indicate the importance of interventions to address household level variables. Such interventions might increase household economic status; e.g., income generation activities, or might improve household water, sanitation, and hygiene conditions. Additionally, we need to consider developing nutrition-sensitive agriculture to improve household food security especially for large-sized households or households with more than two children under five years. Promotion of family planning services is also essential to ensure a sufficiently long, beneficial pregnancy interval [29,30] that will help guarantee the mother has time for adequate care and feeding of all children under five years in the household.

### 5.3. The Role of Maternal and Child’s Health and Adequate Nutrition

Our analyses showed that low birth weight babies had an increased likelihood of being stunted, as found in previous literature [31,32,33,34]. As stunting often begins in utero, the likelihood of being underweight tended to remain until the early childhood stage. Growth of low birth infants was reported to be behind the growth of those with normal weight at birth [35,36]. The child’s sub-optimal growth during the prenatal period is often the result of maternal undernutrition. However, during the postnatal period, optimum feeding practices can mitigate the effects of poor intrauterine growth [32,37]. Thereby, after delivery, if dietary intake is inadequate, aggravated by unhealthy environmental conditions, children will have an increased susceptibility to infections, leading to poor absorption of nutrients and eventually leading to poor growth [37].

We found that children aged 12–23 months had a significantly more increased likelihood of being stunted than those aged <12 months. Other studies also reported that the difference in length between low birth weight and normal weight babies increased with age starting from 12 months until the child reached two years of age [35,36]. The suboptimal growth related to increased age might derive from the challenges associated with the feeding transition from breastfeeding to complementary feeding [37]. Problems with child growth will occur if continued breastfeeding is not accompanied by adequate complementary feeding at the appropriate age. With increased nutritional demand, if a child receives inadequate complementary feeding, impaired linear growth might occur [38,39]. Apart from that, the increased exposure to various childhood diseases and conditions as a result of increased age, such as exposure to poor food hygiene and environmental sanitation, might contribute to poor growth [37].

We also found that boys were more likely to be stunted than girls, and similar findings have been found in previous literature [25,31]. It is postulated that this is a result of the increased vulnerability of boys to infections and other illnesses that can impair child growth [40].

Our findings also indicate that optimum maternal nutrition should be strongly encouraged even before conception, as it is vital to ensure optimum growth in utero [30,41,42]. Trials have examined the benefits of using iron/folic acid supplements or multiple micronutrient supplementation during pregnancy in increasing fetal growth, birth length and postnatal growth [43,44]. There is a strong association between stunting and the consumption of animal-sourced foods, especially multiple types of animal-sourced foods [45]. Furthermore, nutrition education and counseling during pregnancy complemented by nutrition support were found to increase birthweight, which is important for adequate child growth [30,46]. Additionally, education strategies to promote the consumption of macronutrients during pregnancy are needed. The provision of balanced energy protein supplementation particularly among undernourished women can increase fetal growth [47,48].

All of this evidence mirrors the need to promote the utilization of antenatal care services for both mothers and their infants, as found in our analysis. Multiple contacts during antenatal visits lead to regular and repeated contacts with health workers and opportunities for interactive health education sessions. By having adequate antenatal care, mothers would be able to enhance their knowledge of appropriate feeding for their infants after delivery, including breastfeeding and complementary feeding [49]. Mothers could also have the opportunity to receive information about childhood illness and infections, and how to prevent them. Adequate attendance at ANC might also be related to mothers’ attitudes about providing adequate care after delivery, resulting in optimum child growth and well-being.

Our findings indicate the need for further studies with larger sample sizes to identify more potential predictors of childhood stunting in Indonesia. A key area to examine is the role of intergenerational undernutrition, as measured by maternal height, and its association with the risk of childhood stunting. Other areas that need more detailed examination include: at the environmental level, factors such as availability and access to health care facilities or disparities across provinces; at the household level, factors such as access to food sources or household food securities; and at the individual level, maternal health and nutrition status pre-pregnancy and infant and young child feeding practices. Some of these analyses might require pooling the Basic Health Survey data across several survey rounds. This would also grant an opportunity to examine trends in stunting by regions in Indonesia, and trends in risk factors that might account for greater improvements in some provinces.

### 5.4. Strengths and Limitations

One of the strengths of this study is the use of nationally representative data collected by the Ministry of Health with a large sample size that is adequate to analyze the relationships between different levels of variables and stunting in children under two-years-old. Moreover, the multilevel modeling approach used in the analysis allows for the examination of the importance of province, district and cluster on stunting. The use of sampling weight in our analyses could also reduce potential bias. One of the limitations to note in our study was the use of cross-sectional data that does not allow for causal inferences. The information examined in this study depended on the mother’s recall ability. Another limitation is that the selection of variables analyzed depended on their availability in the dataset. There were factors known to be related to stunting such as dietary diversity [22,24], illnesses and infections [31], food security status [23] and short maternal stature [37,50] that were not included in the analysis because they were not available in the dataset released to investigators.

## 6. Conclusions

In summary, our findings indicate the need for integrated interventions to reduce stunting in Indonesia. Interventions should be directed during prenatal and postnatal periods, using multi-sectoral approaches to address various factors from the community to the individual level. There is a strong requirement for efforts to promote adequate dietary intake during pregnancy complemented with educational interventions. It is important to encourage pregnant women to have adequate antenatal care, which will benefit not only mothers but also their children. After delivery, optimal infant and young child feeding practices, from exclusive breastfeeding in the first six months to appropriate complementary feeding, are essential for optimum dietary intake, child growth and development, and to prevent infections and illnesses that can eventually affect growth. Improvement of household economic status, as well as improved water, sanitation and hygiene are also needed. Furthermore, it is essential to ensure the availability and accessibility of safe and healthy food to improve food security in the household.

## Figures and Tables

**Table 1:** Frequency distribution of respondents by different characteristics, The 2013 Indonesia Basic Health Survey.

	All		Stunted		Bivariate Analysis		
	*n*	(%)	*n*	(%)	OR	(95%CI)	*p*
**Region and Place of residence**							
**Region**							
Java and Bali	13,940	(56.5)	4417	(31.7)	1.00		
Sumatera	5445	(22.1)	1942	(35.7)	1.34	(1.13–1.59)	0.001
Nusa Tenggara Bara and Nusa Tenggara Timur (NTB/NTT)	1031	(4.2)	424	(41.1)	1.82	(1.82–2.57)	0.001
Kalimantan and Sulawesi	3526	(14.3)	1255	(35.6)	1.36	(1.36–1.70)	0.007
Maluku and Papua	715	(2.9)	278	(38.9)	1.59	(1.59–1.92)	<0.001
**Place of Residence**							
Urban	12,792	(51.9)	3904	(30.5)	1.00		
Rural	11,865	(48.1)	4412	(37.2)	1.32	(1.18–1.47)	<0.001
**Household and Housing characteristics**							
**Number of household members under five years**							
1	19,132	(77.6)	6364	(33.3)	1.00		
2	5010	(20.3)	1736	(34.6)	1.04	(0.93–1.18)	0.480
3+	515	(2.1)	216	(42.0)	1.37	(1.07–1.75)	0.013
**Number of household members**							
2–4	12,556	(50.9)	4075	(32.5)	1.00		
5–7	10,645	(43.2)	3731	(35.1)	1.13	(1.05–1.21)	0.001
8+	1456	(5.9)	509	(35.0)	1.12	(0.87–1.42)	0.380
**Household wealth index**							
Quintile 1 (Wealthiest)	5016	(20.3)	1441	(28.7)	1.00		
Quintile 2	6397	(25.9)	1944	(30.4)	1.20	(1.08–1.34)	0.001
Quintile 3 (Middle)	5392	(21.9)	1843	(34.2)	1.39	(1.14–1.69)	0.001
Quintile 4	4267	(17.3)	1623	(38.0)	1.66	(1.42–1.94)	<0.001
Quintile 5 (Poorest)	3585	(14.5)	1465	(40.9)	1.79	(1.57–2.05)	<0.001
**Maternal and paternal characteristics**							
**Mother’s education status**							
Academy	2106	(8.5)	612	(29.1)	1.00		
High School	7254	(29.4)	2222	(30.6)	1.07	(0.92–1.23)	0.384
Middle School	6204	(25.2)	2136	(34.4)	1.31	(1.13–1.51)	<0.001
Primary school/no school	8845	(35.9)	3261	(36.9)	1.40	(1.17–1.66)	<0.001
**Father’s education status**							
Academy	1964	(8.0)	565	(28.8)	1.00		
High School	7890	(32.0)	2401	(30.4)	1.07	(0.92–1.24)	0.412
Middle School	5156	(20.9)	1776	(34.5)	1.32	(1.05–1.67)	0.017
Primary school/no school	8315	(33.7)	3113	(37.4)	1.47	(1.24–1.73)	<0.001
**Mother’s employment status**							
Housewife	17,131	(69.5)	5670	(33.1)	1.00		
Working	7278	(29.5)	2560	(35.2)	1.04	(0.96–1.12)	0.323
**Father’s employment status**							
Not working	715	(2.9)	217	(30.4)	1.00		
Working	22,611	(91.7)	7638	(33.8)	1.30	(0.91–1.85)	0.145
**Maternal age at childbirth**							
20–29 years	13,421	(54.4)	4391	(32.7)	1.00		
<20 years	2336	(9.5)	854	(36.6)	1.21	(1.01–1.44)	0.043
30–39 years	7897	(32.0)	2732	(34.6)	1.12	(0.99–1.26)	0.069
>40 years	755	(3.1)	253	(33.6)	1.04	(0.83–1.30)	0.709
**Antenatal care (ANC) services**							
**Number of ANC visits**							
4 or more ANC visits	20,612	(83.6)	6739	(32.7)	1.00		
1–3 ANC visits	2895	(11.7)	1147	(39.6)	1.33	(1.18–1.50)	<0.001
No ANC visit	964	(3.9)	349	(36.2)	1.11	(0.88–1.40)	0.385
**Number of iron/folic acid (IFA) supplements used during pregnancy**							
No IFA used	2469	(10.0)	860	(34.8)	1.00		
<90 IFA used	8629	(35.0)	3050	(35.4)	1.05	(0.87–1.27)	0.606
90 and more IFA used	8661	(35.1)	2735	(31.6)	0.88	(0.71–1.10)	0.278
Do not remember	4898	(19.9)	1671	(34.1)	0.94	(0.75–1.19)	0.627
**Child characteristics**							
**Sex of baby**							
Male	12,323	(50.0)	3897	(31.6)	1.00		
Female	12,334	(50.0)	4419	(35.8)	1.31	(1.21–1.41)	<0.001
**Ever breastfed**							
Ever breastfed	23,148	(93.9)	7793	(33.7)	1.00		
Never breastfed	1509	(6.1)	523	(34.7)	1.11	(0.91–1.35)	0.317
**Child had diarrhea during the last two weeks**							
No	21,851	(88.6)	7339	(33.6)	1.00		
Yes, ≤2 weeks ago	1712	(6.9)	617	(36.0)	1.11	(0.93–1.34)	0.238
Yes, >2 weeks ago	1088	(4.4)	359	(33.0)	1.01	(0.85–1.20)	0.899
**Child Age**							
<12 months	11,749	(47.7)	3331	(28.4)	1.00		
12–23 months	12,908	(52.4)	4985	(38.6)	1.89	(1.53–2.34)	<0.001
**Weight at birth**							
≥2500	15,237	(61.8)	4781	(31.4)	1.00		
<2500	1003	(4.1)	489	(48.8)	2.56	(2.09–3.15)	<0.001
Don’t know	8417	(34.1)	3045	(36.2)	1.30	(1.11–1.51)	0.001
**Age of pregnancy at birth**							
37–40 weeks	10,074	(40.9)	3387	(33.6)	1.00		
<37 weeks	7846	(31.8)	2901	(37.0)	1.21	(1.05–1.40)	0.007
>40 weeks	6733	(27.3)	2028	(30.1)	0.85	(0.72–0.99)	0.039

**Table 2:** Factors associated with stunting among children under two-years-old, The 2013 Indonesia Basic Health Survey.

Variables		Multivariate		Null Model				
	OR	(95%CI)	*p*	OR	(95%CI)			*p*
**Region and Place of residence**								
**Region**								
Java and Bali								
Sumatera	1.29	(1.10–1.50)	0.002					
Nusa Tenggara Barat and Nusa Tenggara Timur	1.50	(1.16–1.93)	0.002					
Kalimantan and Sulawesi	1.24	(1.00–1.53)	0.047					
Maluku and Papua	1.27	(1.05–1.53)	0.013					
**Place of Residence**								
Urban								
Rural								
**Household and Housing characteristics**								
**Household members under five years**								
1	1.00					
2	1.07	(0.94–1.21)	0.312					
3+	1.33	(1.03–1.72)	0.029					
**Number of household members**								
2–4								
5–7	1.11	(1.03–1.20)	0.005					
8+	1.06	(0.83–1.34)	0.641					
**Household wealth index**								
Quintile 1 (Wealthiest)	1.00							
Quintile 2	1.21	(1.09–1.34)	<0.001					
Quintile 3 (Middle)	1.38	(1.14–1.67)	0.001					
Quintile 4	1.63	(1.39–1.89)	<0.001					
Quintile 5 (Poorest)	1.74	(1.51–2.01)	<0.001					
**Maternal and paternal characteristics**								
**Maternal educational status**								
Academy								
High School								
Middle School								
Primary or no schooling								
**Father’s educational status**								
Academy								
High School								
Middle School								
Primary or no schooling								
**Mother’s employment status**								
Housewife								
Working								
Missing								
**Father’s employment status**								
Not working								
Working								
**Maternal age at childbirth**								
<20 years								
20–29 years								
30–39 years								
>40 years								
**Antenatal care (ANC) service**								
**Number of ANC visits**								
4 or more ANC visits	1.00							
1–3 ANC visits	1.22	(1.08–1.39)	0.002					
No ANC visit	0.89	(0.72–1.12)	0.325					
**Number of iron/folic acid (IFA) supplements used during pregnancy**								
No IFA used								
<90 IFA used								
90 and more IFA used								
Do not remember								
**Child characteristics**								
**Sex of baby**								
Female	1.00							
Male	1.33	(1.22–1.45)	<0.001					
**Ever breastfed**								
Never breastfed								
Ever breastfed								
**Child had diarrhea during the last two weeks**								
Yes, ≤2 weeks ago								
Yes, >2 weeks ago								
No								
**Child Age**								
<12 months	1.00							
12–23 months	1.89	(1.54–2.32)	<0.001					
**Weight at birth**								
≥2500 g	1.00							
<2500 g	2.55	(2.05–3.15)	<0.001					
Don’t know	1.13	(0.98–1.30)	0.093					
**Age of pregnancy at birth**								
37–40 weeks								
<37 weeks								
>40 weeks								
Province (MOR)	1.05			1.22				
District (MOR)	1.34			1.32				
Cluster (MOR)	3.31			3.27				
